# Mediator profiles in tears during the conjunctival response induced by allergic reaction in the nasal mucosa

**Published:** 2013-07-12

**Authors:** Zdenek Pelikan

**Affiliations:** Allergy Research Foundation, Breda, Netherlands

## Abstract

**Background:**

The allergic reaction occurring primarily in the nasal mucosa can induce a secondary conjunctival response of an immediate (SICR), late (SLCR), or delayed (SDYCR) type in some patients with allergic conjunctivitis (AC).

**Objectives:**

To investigate the concentration changes of histamine, tryptase, eosinophil cationic protein (ECP), eosinophil-derived neurotoxin (EDN), leukotrienes (LTB _4,_ LTC_4_, LTE_4_), myeloperoxidase (MPO), interferon-γ (IFN-γ), and interleukins (IL-2, IL-4, IL-5) in tears during the SICR, SLCR, and SDYCR.

**Methods:**

In 32 patients with AC, 11 SICR (p<0.01), 13 SLCR (p<0.001), and eight SDYCR (p<0.01) to nasal challenges with allergens (NPTs), the NPTs and 32 control tests with PBS were repeated and supplemented with the determination of these factors in tears.

**Results:**

The SICRs were associated with significant concentration changes in tears (p<0.05) of histamine, tryptase, ECP, LTC_4_, and IL-4. The SLCRs were accompanied by significant changes in concentrations of histamine, ECP, LTB_4_, LTC_4_, MPO, IL-4, and IL-5. The SDYCRs were associated with significant concentration changes in tears (p<0.05) of LTB_4_, MPO, IFN-γ, and IL-2. No significant changes in these factors were recorded in tears during the 32 PBS controls (p>0.1) or in the ten control patients (p>0.1).

**Conclusions:**

These results provide evidence for causal involvement of nasal allergy in some patients with AC, inducing secondary conjunctival response of immediate (SICR), late SLCR, or delayed SDYCR type, associated with different mediator, cytokine, and cellular profiles in the tears, suggesting involvement of different hypersensitivity mechanisms. These results also emphasize the diagnostic value of nasal allergen challenge combined with monitoring of the conjunctival response in some patients with AC.

## Introduction

Allergic conjunctivitis (AC) includes five clinical entities [1-4], a seasonal allergic conjunctivitis, perennial allergic conjunctivitis, vernal keratoconjunctivitis, atopic keratoconjunctivitis, and giant papillary conjunctivitis, all of them having a common causal background, the allergic reaction, but different clinical features. Allergic conjunctivitis (AC) can occur in two forms, a primary and a secondary form, in terms of the locality of the initial allergic reaction [5-10]. In the primary AC form, the initial allergic reaction due to the direct exposure of conjunctivae to an allergen is localized in the conjunctival tissue. In the secondary AC form, the initial allergic reaction taking place in the nasal mucosa, due to exposure to an allergen, subsequently induces secondary AC by factors released during the allergic reaction in the nasal mucosa and reaching the conjunctival tissue through various mechanisms and pathways [5-10].

Various hypersensitivity mechanisms, such as immediate type (IgE-mediated type I), late (type III), or delayed (cell-mediated type IV), may be involved in both forms of AC [1-22]. The involvement of various hypersensitivity types results in the development of various types of conjunctival response (CR) to allergen exposure (challenge), an immediate (ICR), a late (LCR), a dual late (DLCR, a combination of an immediate and a late type), a delayed (DYCR), and a dual delayed (DDYCR, a combination of an immediate and a delayed type) [1-12,15-19,22,23]. The primary forms of AC can be demonstrated by conjunctival provocation tests with allergens (CPTs), whereas the secondary AC forms can be confirmed only by nasal provocation tests with allergens (NPTs) in combination with registration of the conjunctival signs and subjective symptoms. The purpose of this study was to investigate the following: (1) the concentration changes of basic mediators in tears during the secondary immediate (SICR), late (SLCR), and delayed (SDYCR) conjunctival responses; (2) the significance of these mediators and their changes in tears for the mechanism(s) underlying the particular types of secondary conjunctival response.

## Methods

### Patients

Thirty-two of the 81 patients suffering from allergic conjunctivitis, 14 with seasonal allergic conjunctivitis (SAC) and 18 with perennial allergic conjunctivitis (PAC), for more than 3 years, showing insufficient compliance with the standard topical ophthalmologic treatment, referred to our Department of Allergology & Immunology (Institute of Medical Sciences “De Klokkenberg,” Breda, The Netherlands) during 1998–1999 for more extensive analysis of their AC complaints, and developing the secondary conjunctival response (SCR) to nasal provocation tests with allergens (NPTs), volunteered to participate in this study. These patients, 13 men and 19 women, 20–43 years of age (Table 1), had previously been treated with various topical and oral H1-receptor-antagonists, topical cromolyn, glucocorticosteroids, decongestant and vasoconstrictors and incidentally with non-steroidal anti-inflammatory drugs (NSAID), however, with only partial and not fully satisfactory therapeutic effects. None of these patients had other ocular disorders, infection, systemic disease, or immunodeficiency, or had previously been treated with nasal or systemic glucocorticosteroids, nasal cromolyn, or immunotherapy. All of them demonstrated normal intraocular pressure. In 15 of these patients, 19 conjunctival provocation tests (CPTs) with inhalant allergen, performed previously, were negative. The patients underwent a routine diagnostic procedure consisting of a detailed disease history, physical examination, basic laboratory tests, bacteriological screening of tears, nasal secretions, sputum and blood, roentgenogram of chest and paranasal sinuses in Water’s projection, nasoscopy, cytologic examination of nasal secretions, skin tests with inhalant and food allergens, determination of serum immunoglobulins, and ophthalmologic examination including ophthalmoscopy, slit-lamp evaluation, vital staining with fluorescein, and cytologic examination of the tears.

**Table 1:** Characteristics of the patients

	Patients (n=32)			Control subjects n=10
	SICR (n=11)	SLCR (n=13)	SDYCR (n=8)	
Age (years)	25±6	32±11	27±4	30±7
Sex (M/F)	5/6	7/6	3/5	5/5
Disease history (years)	3.1±1.2	3.6±0.9	4.7±1.6	0
Blood leukocyte count (x 10^9^ /l)^*^	8.1±0.9	8.7±1.2	7.5±0.8	7.3±0.6
Blood eosinophil count (x 10^6^ /l) ^**^	305±15	281±20	293±25	266±47
Increased total IgE in the serum ^***^	0	0	0	0
Positive specific IgE in the serum^****^	3	2	1	2
**Positive skin response **^●^				
- immediate type	6	4	0	4
- late type	5	9	2	5
- delayed type	0	0	6	1
**Nasal histamine threshold **^∆^				
- decreased	7	5	1	5
- normal	4	8	7	5
**Nasal provocation tests **^▲^				
- positive	11	13	8	10
- negative	10	14	5	7
**Factors in tears**				
Histamine *ng/ml*	<1.0	<1.0	<1.0	<1.0
Tryptase *µg/l*	<1.0	1.1±0.1	<1.0	<1.0
ECP *µg/l*	2.4±0.2	2.2±0.2	<2.0	<2.0
EDN *ng/ml*	<0.64	<0.64	<0.64	<0.64
LTB4 *pg/ml*	<4.8	<4.8	5.2±0.3	<4.8
LTC4 *pg/ml*	2.3±0.2	2.4±0.3	<2.1	<2.1
LTE4 *pg/ml*	<3.7	<3.7	<3.7	<3.7
MPO *ng/ml*	<25.0	<25.0	<25.0	<25.0
IFN-γ *pg/ml*	<1.0	<1.0	1.4±0.2	<1.0
IL-2 *pg/ml*	<3.0	<3.0	<3.0	<3.0
IL-4 *pg/ml*	1.0±0.3	0.8±0.1	<0.7	<0.7
IL-5 *pg/ml*	<0.32	3.4±0.2	<0.32	<0.32

^*^=normal value 4.0–10.0×10^9^/L; ^**^=normal value <300×10^6^/l; ^***^=normal value <500 IU/ml; ^****^=normal value <0.70 U/ml; ^●^=positive skin response to the relevant allergen (=allergen producing positive nasal and conjunctival or nasal response only); ^∆=^ Normal value >8.0 mg/ml; ^▲^=single determination during the routine diagnostic procedure

The routine diagnostic procedure performed in these 32 patients revealed positive or suspect history for nasal allergy (93%), positive skin tests with various inhalant allergens (100%), hyperaemic /livid and edematic nasal mucosa (97%), increased eosinophil and neutrophil counts in nasal secretions (87%), conjunctival hyperaemia and tearing to a slight degree (100%), appearance of incidental eosinophils and conjunctival epithelial cells in the tear specimens (78%), increased blood eosinophil counts (22%), and positive specific IgE in the serum (ImmunoCAP) for some inhalant allergens (19%; Table 1). In these 32 patients, 61 NPTs with inhalant allergens (Table 2) and 32 control challenges with phosphate-buffered saline (PBS; 8 g NaCl, 0.2 g KCl, 1.15 g Na_2_HPO_4_ , 0.2 g KH_2_ PO_4_, distilled water ad 1000 μl; pH=7.4) were performed using rhinomanometry combined with simultaneous recording of the ocular signs and subjective symptoms.

**Table 2:** Survey of the allergens used for nasal challenge.

Allergen	Concentration BU/ml	Nasal responses positive (n=32)	Conjunctival responses			Nasal responses negative (n=29)
			SICR (n=11)	SLCR (n=13)	SDYCR (n=8)	
Dermatophagoides pteronyss.	1000	4	1	2	1	3
Dermatophagoides farinae	1000	1	0	1	0	1
**Animal danders**						
- dog	3000	2	0	1	1	2
- horse	3000	1	1	0	0	0
- cat	2000	3	2	0	1	2
- guinea pig	2000	1	0	1	0	0
- hamster	2000	2	1	1	0	1
**Feathers**						
- parrot	3000	1	1	0	0	1
- parakeet	3000	1	0	1	0	0
- pigeon	2000	1	0	1	0	2
Aspergillus fumigatus	1000	1	0	0	1	1
**Pollen**						
- grass mix I	1000	3	2	1	0	4
- grass mix II	1000	2	0	1	1	2
- flower mix	5000	1	1	0	0	3
- tree mix	3000	2	1	0	1	2
- weed mix	1000	1	0	0	1	0
- poplar	2000	1	0	1	0	1
- birch	1000	2	1	1	0	2
- ragweed short	1000	1	0	0	1	1
- ragweed giant	1000	1	0	1	0	1

BU/ml=biologic units per mL Grasspollen mix I=*Dactylis glomerata, Lolium perenne, Phleum pratensis, Poa pratensis;* Grasspollen mix II=*Festuca pratensis, Holcus lanatus, Agrostis alba, Anthoxanthum odoratum.* Flower pollen mix=*Dahlia variabilis, Solidago virgaurea, Primula variabilis, Forsythia suspense.* Tree pollen mix=*Betula pendula, Corylus avellana, Juniperus communis, Salix alba*. Weed pollen mix=*Artemisia vulgaris,Plantago lanceolata, Rumex acetosa, Taraxacum officinale*

The ocular signs and relevant subjective symptoms were evaluated with Pelikan’s scoring (grading) system [7-10]. The patients were investigated in a period without acute ocular and nasal complaints, during hospitalization. The long-acting H1-receptor antagonists, topical cromolyn and glucocorticosteroids, were withdrawn 4 weeks, whereas other treatments were withdrawn 48 h before each NPT. The 32 positive NPTs producing an SCR of any type and 32 PBS control challenges were repeated 2–3 weeks later. The repeated NPTs and PBS controls were supplemented with tear collection for the mediator determination. A 4-day interval was always inserted between the end of the preceding test and the beginning of the following test to prevent carry-over effects and to allow for patient to recover. The study protocol was approved by the local ethical committee (IRB-MCK), and informed consent was obtained from all study participants. The study has been performed according to the WMA Declaration of Helsinki concerning the principles for medical research involving human subjects.

### Allergens

Dialyzed and lyophilized allergen extracts (Allergopharma, Reinbek, Germany) were diluted in PBS and used for skin tests in concentrations of 100–500 BU/ml and for NPTs in concentrations of 1000–5000 BU/ml (Table 2), as recommended by the manufacturer.

### Skin tests

Skin prick tests (SPTs) with allergenic extracts in concentrations of 500 BU/ml were performed and evaluated after 20 min. The intracutaneous tests in concentrations of 100 BU/ml and 500 BU/ml were then performed in all patients and evaluated 20 min and 6, 12, 24, 36, 48, 56, 72, and 96 h after the intradermal injection. A skin wheal (>7.0 mm in diameter) occurring after 20 min was qualified as a positive immediate skin response, skin infiltration appearing between 6 and 12 h as a late skin response, and skin induration recorded later than 48 h as a delayed skin response [6-10].

### Nasal provocation tests

**Figure 1:**
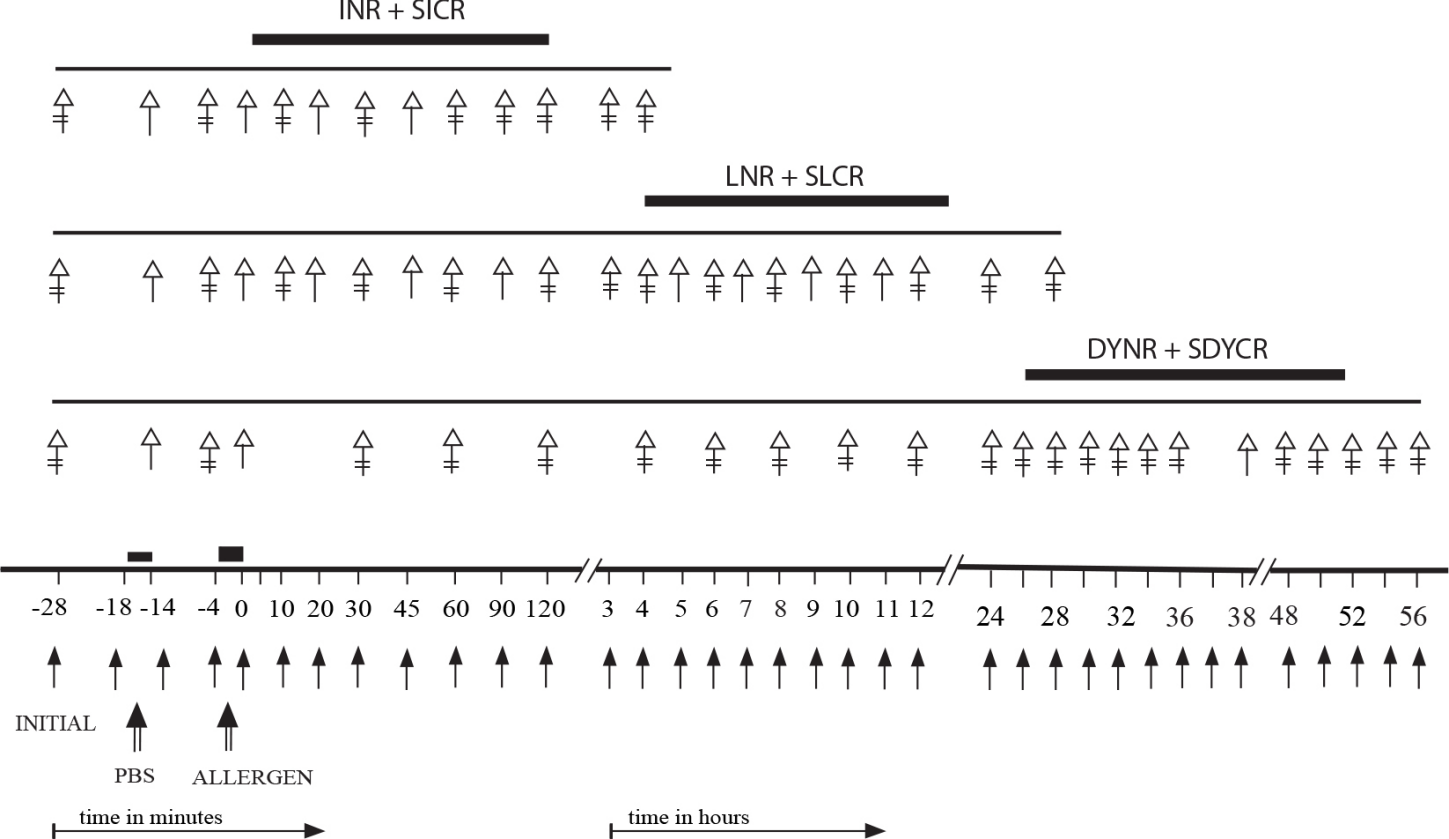
The schedule of the nasal provocation tests with allergens combined with recording of ocular features and collection of the tear samples. PBS= control nasal challenge with phosphate buffered saline; ALLERGEN= nasal challenge with allergen; INR + SICR= Immediate nasal and secondarily induced conjunctival responses; LNR + SLCR = Late nasal and secondarily induced conjunctival responses; DYNR + SDYCR =Delayed nasal and secondarily induced conjunctival responses; Arrow with solid point= recording of the NPG values (rhinomanometric parameters); Arrow with hollow point = recording of the objective ocular signs and subjective symptoms; Double crossed arrow = collection of the tear samples.

Nasal challenges with allergens were performed using rhinomanometry, described in our previous studies [5-10,24]. Nasal obstruction due to the nasal mucosa edema was evaluated by nasopharynx-nostril pressure gradient (NPG) parameters, which are the pressure differences (∆P) between the nasopharyngeal cavity and the outside air, expressed in cm H_2_O. NPTs were performed using the following schedule: (1) baseline values recorded at 0, 5, and 10 min; (2) PBS control values recorded at 0, 5, and 10 min after a 3-min application of PBS to the nasal mucosa of the non-intubated nasal cavity by a saturated wad of cotton wool on a nasal probe inserted under the middle turbinate; (3) the post-challenge values recorded after a 3-min challenge with the allergen, performed in the same manner as the challenge with PBS, at 0, 5, 10, 20, 30, 45, 60, 90, and 120 min, then every hour up to the 12^th^ h, and every second hour during the time periods between the 24^th^–38^th^ and 48^th^–56^th^ (60^th^) h (Figure 1) [5-10,24]. The allergens used for the NPTs were chosen with respect to the disease history and positive skin tests (Table 1 and Table 2).

The nasal response was assessed as positive when the post-challenge mean NPG values increased by at least 2.0 cm H_2_O (1.2±0.3, mean±standard error [SE]) regarding the baseline values, recorded at least at three consecutive time intervals [5-10,24]. The NPG changes recorded within 60–120 min after the allergen challenge were considered to be an immediate (INR), those recorded within 4–12 h to be a late (LNR), and the changes measured later than 24 h to be a delayed (DYNR) response [5-10,24].

### Control challenge with phosphate-buffered saline

The control nasal challenge with PBS was performed in each patient studied by the same schedule as that used for the NPTs with allergen.

### Conjunctival response

The objective conjunctival signs and subjective symptoms were registered before and during all NPTs with allergens and PBS at the same time points as the nasal NPG values (Figure 1). The features of the conjunctiva were assessed by ophthalmoscopy including Pelikan’s grading scale [2,7-10,25-27]. The Abelson’s grading scale included either conjunctival hyperemia and itching only [26] or hyperemia, chemosis, tearing, and itching [25,27] evaluated with three [25,27] or four [26] grades (0=none; 1=mild [intermittent]; 2=moderate; 3=severe; 4=extremely severe [or “incapacitating” itching]; significant threshold: ≥+2). Our (Pelikan’s) modified grading scale included not only hyperemia, chemosis, hyperlacrimation, and itching, but also palpebral edema, photophobia, and blurred vision, evaluated with a four-grade system (0=absent; 1=mild [present to a slight degree or intermittent]; 2=moderate; 3=pronounced [moderately severe]; 4=severe). Differences in a total sign score of 4 or more points (3±1, mean±SE), regarding the prechallenge value, recorded at least at three consecutive time intervals, were statistically significant (p<0.05) [5,7-9].

### Collection and processing of tears for mediator measurements

The tear specimens (80–150 μl) were gently collected with a sterilized micropipette from each eye separately from the inferior conjunctival fornix and/or lacus lacrimalis at the following time intervals: (a) at 10, 5, and 0 min before the NPT and (b) at 0, 5, 10, 20, 30, 60, and 120 min and every second hour up to 12 h and between the 24^th^–38^th^ and 48^th^–56^th^ (60^th^) h after the allergen challenge (Figure 1). The tear specimens collected before the NPT, those collected up to 10 min, and those obtained at 20 and 30 min after the NPT were pooled to obtain sufficient material quantity. Care was taken to avoid touching the eye lid margins, corneal surface, and conjunctiva, as far as possible. Immediately after collection, the tear samples were divided into two equal portions; one portion was processed for cytologic examination, whereas the other portion was centrifuged at 1000 × *g* for 1 min at 4^○^ C. The supernatants were removed and stored at −8 °C.

The factors in tears were measured by commercial kits, according to the manufacturer’s recommendations. The measurements were performed in tear samples from each eye separately on each occasion, in duplicate and by a double-blind schedule. The results were then calculated as the mean of both eyes. The intra-assay and the inter-assay coefficients of variations for the assay kits were less than 10%. The detection limits measured by the author are abbreviated as DL. The following factors were recorded: (a) Histamine concentrations, so-called blanks, were measured by Siraganian’s fluorometric method [28], DL: 1.0 ng/ml; (b) Tryptase *-*ImmunoCAP (Pharmacia Diagnostics, Uppsala, Sweden), DL: 1.0 μg/L; (c) Eosinophil cationic protein (ECP) –ImmunoCAP (Pharmacia), DL: 2 μg/l; (d) Eosinophil-derived neurotoxin (EDN/EPX)- enzyme-linked immunoassay (ELISA) kit (MBL International Corp., Woburn, MA), DL: 0.64 ng/ml; (e) Leukotrienes B4, C4, E4 -EIA kits (Cayman Chemical Company, Ann Arbor, MI), DL: LTB_4_=4.8 pg/ml, LTC_4_=2.1 pg/ml, LTE_4_=3.7 pg/ml; (f) Myeloperoxidase (MPO) *-*ELISA kit (Oxis International Inc., Portland, OR), DL: 25 ng/ml; (g) Interferon-gamma (IFN-γ)-ELISA kit (Bender MedSystems, Wien, Austria), DL:1.0 pg/ml; (h) Interleukin 4 (IL-4) *-* ELISA kit (Bender), DL: 0.7 pg/ml; (i) Interleukin 2 (IL-2) - ELISA kit (R & D System, Minneapolis, MN), DL:<3.0 pg/ml; (j) Interleukin 5 (IL-5) - ELISA kit (R & D System), DL: 3.2 pg/ml.

### Collection and processing of tears for the additional cytologic examination

The tear specimens were spread out on the slide surface with a glass probe, air-dried, fixed with polyethylene glycol, and stained with May-Grünwald-Giemsa, which we modified [7,23,29,30]. Specimens were then dehydrated with methyl alcohol, mounted in Canada balsam, and scanned microscopically in a double-blind manner [7,23,29,30]. The particular cell types were distinguished according to the cytologic criteria described in detail in our previous papers [7,23,29,30]. The absolute numbers of particular cell types were counted per microscopic field at magnification 250x and the mean values calculated from 20 fields, per each eye separately. The mean values from both eyes were finally calculated and expressed in numbers of cells per 1 microscopic field (250x magnification). Doubtful cells were reexamined under oil immersion (magnification 1200x).

### Control group

Ten randomly selected adults suffering from allergic rhinitis without history of ocular disease and with normal ophthalmologic findings volunteered to participate as control subjects. In these patients, 10 positive NPTs with inhalant allergens (four INR, four LNR, two DYANR) were repeated and supplemented with registration of the conjunctival and subjective symptoms and a single determination of these mediators in tears (Table 1).

### Statistical analysis

(1) Nasal and ocular responses (mean total signs and symptom scores) and the PBS control challenges in individual patients were analyzed by the Wilcoxon matched-pair signed rank test, comparing the post-challenge values at each time point with the mean prechallenge values. (2) The mean NPG values and the mean total ocular score values were compared with the corresponding PBS control values at each time point and analyzed by the Mann–Whitney U test. (3) The post-challenge mediator values measured at each time point during the repeated NPT and PBS controls in each patient were compared with their prechallenge values and analyzed by the Wilcoxon matched-pair signed rank test. (4) The mean post-challenge mediator values were also compared with corresponding PBS values and evaluated by the Mann–Whitney U test. Statistical evaluation was performed separately for each eye, and then the mean values from both eyes were calculated. A p value <0.05 was considered statistically significant.

## Results

### Nasal responses

The 32 patients in whom 61 NPTs with various inhalant allergens (Table 2) and 32 PBS control challenges were performed, developed 32 positive nasal responses, 11 immediate (INR; p<0.01), 13 late (LNR; p<0.001), eight delayed (DYNR; p<0.05), and 29 negative nasal responses (NNRs; p>0.1; Table 2). The 32 PBS control tests were negative (p>0.1).

**Figure 2:**
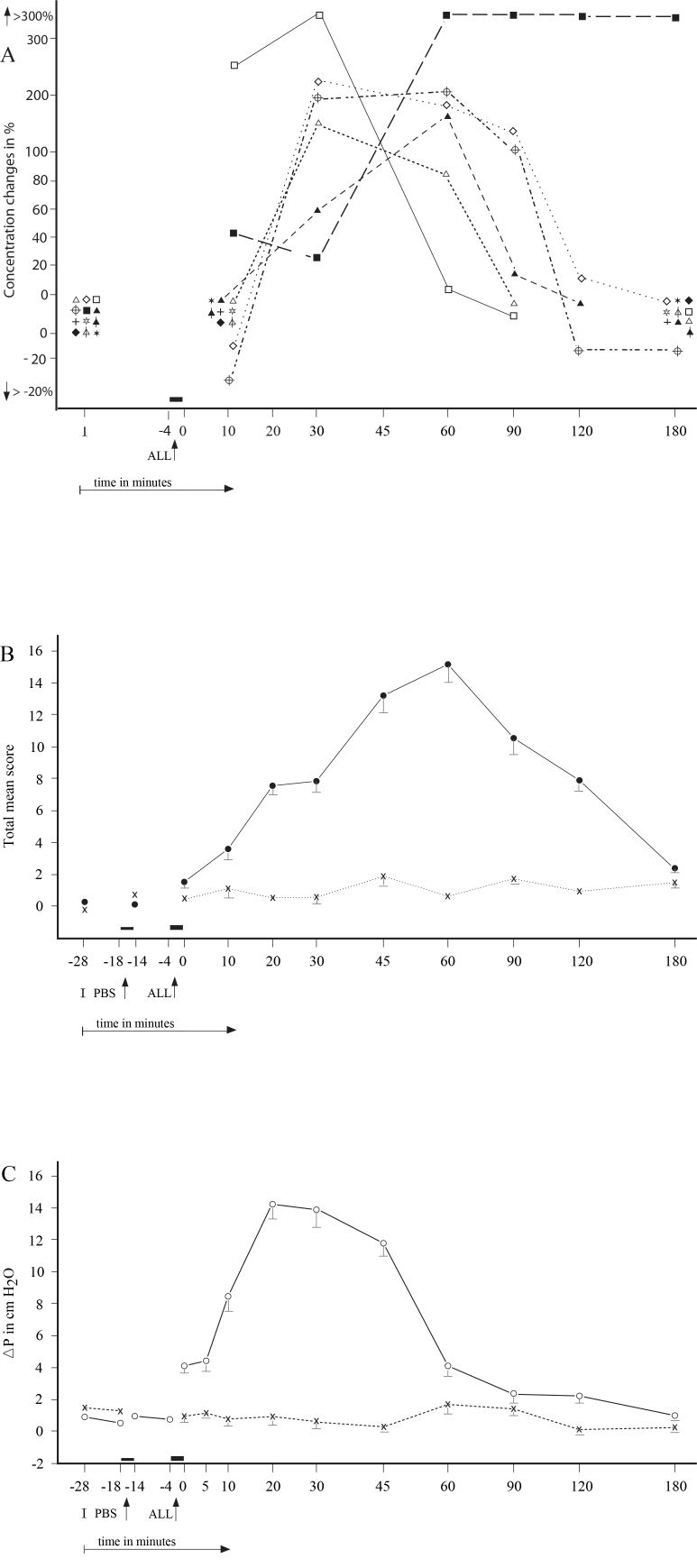
The secondary immediate ocular (conjunctival + corneal) responses (SIOR) were recorded in 11 patients. **A**: The mean score of particular factors recorded during the SIOR: hollow square= Histamine; hollow triangle = Tryptase; crossed circle= ECP; hollow star = EDN; hollow triangle with vertical line = LTB4 ; hollow rhombus = LTC4 ; cross = LTE4 ; solid star = MPO; solid rhombus = IFN-γ; solid triangle with vertical line= IL-2; solid square= IL-4; solid triangle= IL-5. **B**: The total mean score of ocular signs and symptoms recorded during the SIOR (●) and PBS (x). **C**: The mean rhinomanometric values (NPG) recorded during immediate nasal response to allergen challenge (○) and control challenge with phosphate-buffered saline (PBS, x); I = Initial (baseline) values; ALL= Nasal challenge with allergen.

**Figure 3:**
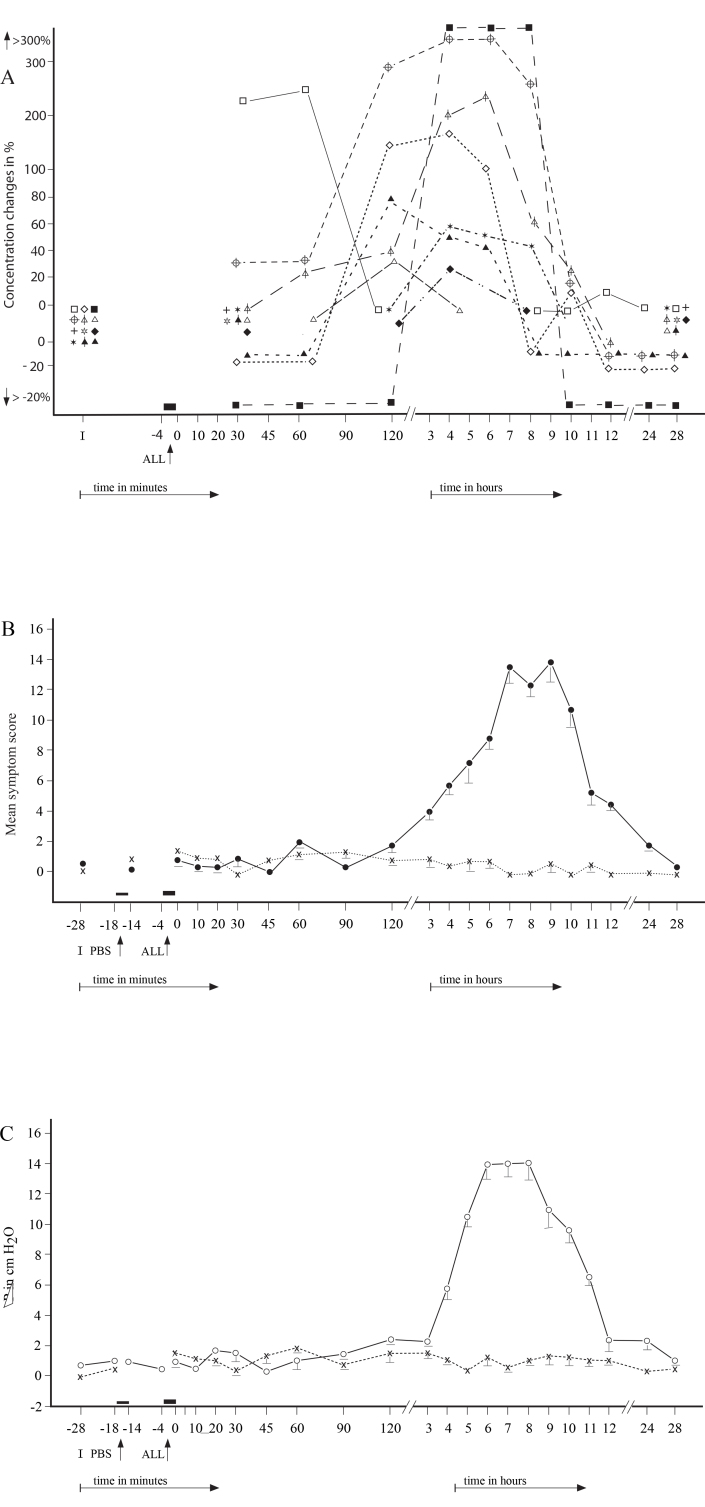
The secondary late ocular (conjunctival + corneal) responses (SLOR) were recorded in 13 patients.** A**: The mean score of particular factors recorded during the SLOR: hollow square= Histamine; hollow triangle = Tryptase; crossed circle= ECP; hollow star = EDN; hollow triangle with vertical line = LTB4; hollow rhombus = LTC4 ; cross = LTE4 ; solid star = MPO; solid rhombus = IFN-γ; solid triangle with vertical line= IL-2; solid square = IL-4; solid triangle = IL-5. **B**: The total mean score of ocular signs and symptoms recorded during the SLOR (●) and PBS (x). **C**: The mean rhinomanometric values (NPG) recorded during late nasal response to allergen challenge (○) and control challenge with phosphate-buffered saline (PBS, x). I = Initial (baseline) values; ALL = Nasal challenge with allergen.

**Figure 4:**
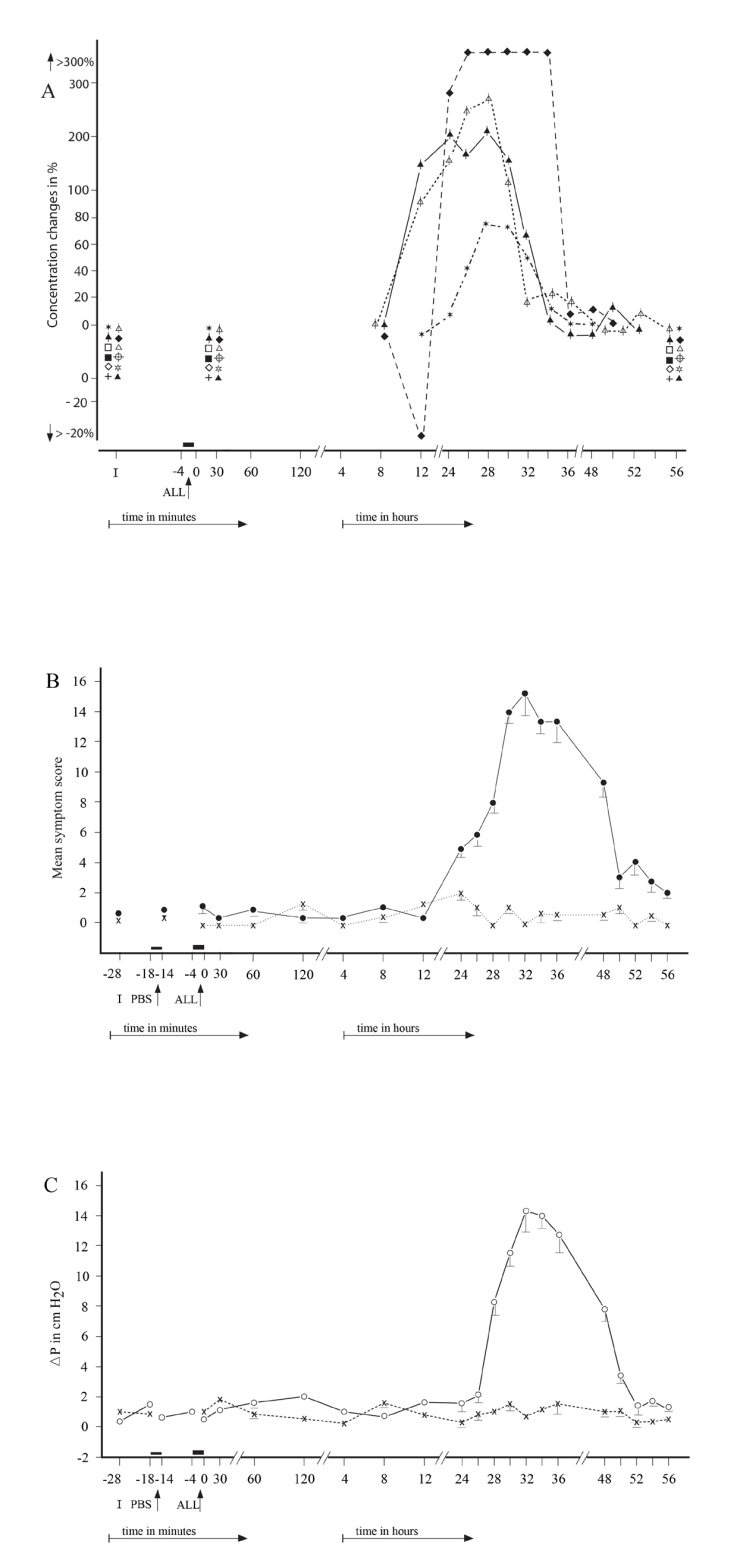
The secondary delayed ocular (conjunctival + corneal) responses (SDYOR) were recorded in 8 patients. **A**:****The mean score of particular factors recorded during the SDYOR: hollow square= Histamine; hollow triangle = Tryptase; crossed circle= ECP; hollow star = EDN; hollow triangle with vertical line = LTB_4_; hollow rhombus = LTC_4_; cross = LTE_4 _; solid star = MPO; solid rhombus = IFN-γ; solid triangle with vertical line= IL-2; solid square = IL-;, solid triangle= IL-5. **B**:****The total mean score of ocular signs and symptoms recorded during the SDYOR (●) and PBS (**x**).** C**:****The mean rhinomanometric values (NPG) recorded during delayed nasal response to allergen challenge (○) and control challenge with phosphate-buffered saline (PBS, x). I = Initial (baseline) values; ALL = Nasal challenge with allergen.

No significant differences were found in the appearance of the particular NR types regarding the individual allergens (p>0.1). The repeated NPTs resulted in the development of similar NR types (Figure 2C, Figure 3C, Figure 4C). No statistical significant differences were found between the initial and repeated responses (p>0.2).

### Conjunctival responses

The 32 positive NRs were associated with significantly positive secondary conjunctival responses (SCR), 11 immediate (SICR; p<0.01), 13 late (SLCR; p<0.01), and eight delayed (SDYCR; p<0.05), whereas no CR was observed during the 29 NNRs (p>0.2). The SCRs were represented by significant changes in the objective conjunctival signs (p<0.01) as well as subjective symptoms (p<0.05). The 32 repeated NPTs induced similar and statistically significant SCRs, when the post-challenge values were compared with the prechallenge (baseline) values (p<0.01) and with the PBS control challenge (p<0.01). (Figure 2B, Figure 3B, Figure 4B).

No significant differences in the conjunctival features recorded during the initial and the repeated SCRs were observed between the right and left eye (p>0.1). No significant corneal signs were recorded in any SCR type. No conjunctival changes were recorded during the 32 PBS control challenges (p>0.1).

### Changes in mediators and other factor in the tears during the secondary conjunctival responses

The SICRs were associated with significant concentration changes (p<0.05) of histamine, tryptase, ECP, LTC_4_, and IL-4 in the tears (Table 3; Figure 2A), whereas the concentrations of EDN, LTB_4_, LTE_4_, MPO, IFN-γ, and IL-2 were either under the detection limits or unchanged. The SLCRs were accompanied by significant changes in the concentrations (p<0.05) of histamine, ECP, LTB_4_, LTC_4_, MPO, IL-4, and IL-5 (Table 4; Figure 3A), but the concentrations of tryptase, EDN, LTE_4_, IFN-γ, and IL-2 were under the detection limits or unchanged. The SDYCRs were associated with significant concentration changes (p<0.05) of LTB_4_, MPO, IFN-γ, and IL-2 in the tears (Table 5; Figure 4A), while the concentrations of histamine, tryptase, ECP, EDN, LTC_4_, LTE_4_, IL-4, and IL-5 were under the detection limits or unchanged, The prechallenge concentrations of most factors were either low or under the detection limit (Table 1), whereas the post-challenge concentration changes appeared during the particular SCR types. No significant concentration changes in these factors were recorded in tears during the 32 PBS controls or 29 negative CRs. No significant differences in the concentration changes of the particular factors in tears were found between the right and left eye, during the SCRs and during the PBS controls (p>0.1 and p>0.2, respectively).

**Table 3:** Concentration of particular factors in tears during the secondary immediate conjunctival response (SICR; n=11).

Factors	Before the challenge	After the challenge (min)						
		0–10	20–30	60	90	120	180	240
**Histamine *ng/ml***								
- SICR	<1.0	3.5±0.9*	5.1±1.3*	1.2±0.4	<1.0	<1.0	<1.0	<1.0
- PBS	<1.0	<1.0	1.2±0.1	<1.0	<1.0	<1.0	<1.0	<1.0
**Tryptase *µg/l***								
- SICR	<1.0	<1.0	2.7±0.4*	1.9±0.6*	<1.0	<1.0	<1.0	<1.0
- PBS	<1.0	<1.0	<1.0	<1.0	<1.0	<1.0	<1.0	<1.0
**ECP *µg/l***								
- SICR	2.3±0.1	<2.0	6.8±1.3*	7.2±0.8*	4.5±0.7*	<2.0	<2.0	<2.0
- PBS	2.1±0.1	<2.0	<2.0	<2.0	2.1±0.1	<2.0	<2.0	<2.0
**LTC4 *pg/ml***								
- SICR	2.3±0.1	<2.1	6.7±0.9*	6.3±1.0*	5.2±0.8*	2.6±0.3	<2.1	<2.1
- PBS	2.2±0.1	<2.1	<2.1	<2.1	2.3±0.2	<2.1	<2.1	<2.1
**IL-4 *pg/ml***								
- SICR	0.9±0.2	1.3±0.5	1.1±0.4	4.0±0.5*	7.7±1.2*	10.5±2.1*	8.4±0.6*	<0.7
- PBS	1.3±0.4	<0.7	<0.7	1.4±0.3	<0.7	<0.7	<0.7	<0.7
**IL-5 *pg/ml***								
- SICR	<3.2	<3.2	3.8±0.5	3.3±0.1	3.4±0.2	<3.2	<3.2	<3.2
- PBS	<3.2	<3.2	<3.2	<3.2	<3.2	<3.2	<3.2	<3.2

SICR=Secondary immediate conjunctival response; PBS=Phosphate-buffered saline; Significance with respect to the baseline (before the challenge): *=p<*0.05*;

**Table 4:** Concentration of particular factors in tears during the secondary late conjunctival response (SLCR; n=13).

Factors	Before the challenge	After the challenge (h)									
		1/2	1	2	4	6	8	10	12	24	28
**Histamine* ng/ml***											
- SLCR	<1.0	<1.0	3.3±0.5*	4.9±1.2*	3.5±0.7*	<1.0	<1.0	<1.0	1.1±0.1	<1.0	<1.0
- PBS	<1.0	<1.0	<1.0	<1.0	<1.0	<1.0	<1.0	<1.0	<1.0	<1.0	<1.0
**ECP* µg/l***											
- SLCR	2.3±0.1	3.0±0.7	8.8±1.3*	9.2±0.8*	12.5±0.7*	9.9±1.0*	9.3±1.4*	3.0±0.5	<2.0	<2.0	<2.0
- PBS	2.5±0.4	2.1±0.1	<2.0	<2.0	<2.0	<2.0	<2.0	<2.0	<2.0	<2.0	<2.0
**LTB4* pg/ml***											
- SLCR	<4.8	<4.8	5.8±1.3	9.4±0.7*	16.6±1.8*	14.9±1.1*	8.5±1.2*	6.1±0.7	<4.8	<4.8	5.2±0.3
- PBS	<4.8	<4.8	<4.8	<4.8	<4.8	<4.8	<4.8	<4.8	5.1±0.2	<4.8	<4.8
**LTC4* pg/ml***											
- SLCR	2.4±0.3	<2.1	<2.1	5.5±0.4*	6.0±1.2*	5.9±0.8*	2.2±0.1	2.6±0.3	<2.1	<2.1	<2.1
- PBS	2.2±0.1	<2.1	<2.1	<2.1	<2.1	2.3±0.2	<2.1	<2.1	<2.1	<2.1	<2.1
**MPO* ng/ml***											
- SLCR	<25.0	<25.0	<25.0	<25.0	39.4±3.0*	48.1±2.6*	45.9±4.2*	<25.0	<25.0	<25.0	<25.0
- PBS	<25.0	<25.0	<25.0	<25.0	<25.0	<25.0	<25.0	<25.0	<25.0	<25.0	<25.0
**IL-4* pg/ml***											
- SLCR	1.3±0.4	<0.7	<0.7	<0.7	6.2±1.3*	8.9±0.6*	5.1±0.2*	<0.7	<0.7	<0.7	<0.7
- PBS	0.8±0.1	<0.7	<0.7	<0.7	<0.7	<0.7	1.0±0.3	<0.7	<0.7	<0.7	<0.7
**IL-5* pg/ml***											
- SLCR	3.5±0.3	<3.2	<3.2	7.1±0.8*	6.5±0.6*	4.9±0.3	<3.2	<3.2	<3.2	<3.2	<3.2
- PBS	<3.2	<3.2	<3.2	<3.2	<3.2	<3.2	<3.2	<3.2	<3.2	<3.2	<3.2

SLCR=Secondary late conjunctival response; PBS=Phosphate-buffered saline; Significance with respect to the baseline (before the challenge): *=p<*0.05*

**Table 5:** Concentration of particular factors in tears during the secondary delayed conjunctival response (SDYCR; n=8).

Factors	After the challenge (h)										
	Before the challenge	12	24	26	28	30	32	34	36	48	56
**LTB4* pg/ml***											
-SDYCR	<4.8	9.1±1.4*	22.9±2.8*	27.0±1.5*	14.3±2.2*	19.7±2.1*	9.5±1.3*	6.4±2.0	5.5±0.6	<4.8	<4.8
-PBS	<4.8	<4.8	<4.8	<4.8	<4.8	<4.8	<4.8	<4.8	5.3.±0.4	<4.8	<4.8
**MPO* ng/ml***											
-SDYCR	<25.0	<25.0	43.5±3.9*	38.2±4.1*	46.7±3.3*	42.0±2.7*	39.3±1.9*	27.6±1.1	25.8±0.5	<25.0	<25.0
-PBS	<25.0	<25.0	<25.0	<25.0	<25.0	<25.0	<25.0	<25.0	27.4±1.0	<25.0	<25.0
**IFN-γ* pg/ml***											
-SDYCR	1.6±0.4	<1.0	16.7±3.4*	42.9±2.3*	37.5±1.8*	33.0±2.2*	24.5±1.8*	11.0±0.6*	8.8±1.9*	<1.0	<1.0
-PBS	1.2±0.1	<1.0	<1.0	<1.0	<1.0	<1.0	<1.0	<1.0	<1.0	<1.0	<1.0
**IL-2 pg/ml**											
-SDYCR	<3.0	7.4±1.3*	9.5±0.7*	10.2±1.4*	9.1±0.6*	8.0±1.1*	3.4±0.3	3.3±0.2	<3.0	<3.0	<3.0
-PBS	<3.0	<3.0	<3.0	<3.0	<3.0	<3.0	<3.0	<3.0	<3.0	<3.0	<3.0

SDYCR=Secondary delayed conjunctival response; PBS=Phosphate-buffered saline; Significance with respect to the baseline (before the challenge): *=p<*0.05*;

### Cytologic changes in tears during the particular secondary conjunctival response types

The prechallenge cell counts in tears were low compared with the primary types of AC [5,7] The SICR was associated with increased counts of eosinophils and mast cells in tears between 30 and 60 min and of the epithelial cells between 60 and 90 min after the NPT (Table 6). The SLCR was accompanied by increased counts of eosinophils between 4 and 8 h, neutrophils between 6 and 9 h, basophils between 3 and 4 h, lymphocytes between 10 and 11 h, goblet cells at 10 h, and epithelial cells between 7 and 9 h after the NPT (Table 7). The SDYCR was associated with increased counts of neutrophils between 24 and 32 h, lymphocytes between 24 and 34 h, monocytes between 12 and 30 h, goblet cells between 34 and 36 h, and epithelial cells between 26 and 34 h after the NPT (Table 8). No significant cellular changes were found in tears of the 32 PBS controls, and only sporadic epithelial cells were recorded in tears during the 29 NNRs. No significant differences were found in results between both the eyes.

**Table 6:** Mean numbers of particular cell types in tears (per microscopic field at 250×magnification) during the positive SICR and PBS challenge (n=11).

Cells	Before the challenge	After the challenge (min)							
		0–10	20–30	45	60	90	120	180	240
**Eosinophils**									
-SICR	1	3*	5*	4*	3*	0.5	0	0	0.5
-PBS	0.5	0	0	0	0	0	0.5	0	0.5
**Neutrophils**									
- SICR	0	0	0	1	1	0	0	0	0
-PBS	0	0	0	0	0	0	0	0	0
**Basophils**									
-SICR	0	0	0	0	0	0	0	0	0
-PBS	0	0	0	0	0	0	0	0	0
**Mast cells**									
-SICR	0	0.5*	1.5*	1*	0	0	0	0	0
-PBS	0	0	0	0	0	0	0	0	0
**Lymphocytes**									
-SICR	0	0	0	0	0	0	0	0	0
-PBS	0	0	0	0	0	0	0	0	0
**Monocytes**									
-SICR	0	0	0	0.15	0	0	0	0	0
-PBS	0	0	0	0	0	0	0	0	0
**Goblet cells**									
-SICR	0	0	0	0	0.1	0	0	0	0
-PBS	0	0	0	0	0	0	0	0	0
**Epithelial cells**									
-SICR	0	0	0	0	2.5*	2.0*	0.5	0	0
-PBS	0	0	0	0	0	0	0	0	0

SICR=Secondary immediate conjunctival response; PBS=Phosphate-buffered saline; Significance with respect to the baseline (before the challenge): ^+^=p≤*0.05;* *=p<*0.05*; The statistically significant magnitude of changes in the cells in tears (mean ± SD), were as follows: eosinophils 2 (2.10±0.14); neutrophils 2 (2.25±0.21); basophils 0.3 (0.28±0.04); mast cells 0.5 (0.35±0.11); lymphocytes 1 (1.23±0.16); monocytes 0.4 (0.41±0.19); epithelial cells 2 (2.17±0.41); goblet cells 0.2 (0.20±0.04) [5,7,8,29,30,56].

**Table 7:** Mean numbers of particular cell types in tears (per microscopic field at 250×magnification) during the positive SLCR and PBS challenge (n=13).

Cells	Before the challenge	After the challenge (h)														
		1/2	1	2	3	4	5	6	7	8	9	10	11	12	24	28
**Eosinophils**																
-SICR	0.5	1	0.5	0	1	4*	5*	4*	4.5*	3*	0.5	0	0	0	0.5	0.5
-PBS	0.5	0	0	0	0	0	0	0	0	0	0	0	0	0.5	0.5	0.5
**Neutrophils**																
-SICR	0.2	0	0	0	0	0	1	4*	4*	3*	2*	0	0	0	0	0.1
-PBS	0	0.3	0	0	0	0	0	0	0.3	0.1	0	0	0	0	0	0
**Basophils**																
-SICR	0	0	0	0.2	0.5*	0.3*	0.1	0	0	0	0	0	0	0	0	0
-PBS	0	0	0	0	0	0	0	0	0	0	0	0	0	0	0	0
**Mast cells**																
-SICR	0	0.1	0	0	0	0	0	0	0	0	0	0	0	0	0	0
-PBS	0	0	0	0	0	0	0	0	0	0	0	0	0	0	0	0
**Lymphocytes**																
-SICR	0	0	0	0	0	0	0	0	0	0	0	1*	1*	0.5	0	0
-PBS	0	0	0	0	0	0	0	0	0	0	0	0	0	0	0	0
**Monocytes**																
-SICR	0	0	0	0	0	0	0	0.1	0.1	0	0	0	0	0	0	0
-PBS	0	0	0	0	0	0	0	0	0	0	0	0	0	0	0	0
**Goblet cells**																
-SICR	0	0	0	0	0	0	0	0	0	0	0	0.3*	0	0	0	0
-PBS	0	0	0	0	0	0	0	0	0	0	0	0	0	0	0	0
**Epithelial cells**																
-SICR	1	0	0	0	0	0	0.2	0.5	2.5*	3*	2.5*	0.4	0	0	0	0
-PBS	1	0	0	0.5	0	0	0	0	0	0	0	0	0	0	0.2	0

SLCR=Secondary late conjunctival response; PBS=Phosphate-buffered saline; Significance with respect to the baseline (before the challenge): ^+^=p≤*0.05;* *=p<*0.05*; The statistically significant magnitude of changes in the cells in tears (mean ± SD), were as follows: eosinophils 2 (2.10±0.14); neutrophils 2 (2.25±0.21); basophils 0.3 (0.28±0.04); mast cells 0.5 (0.35±0.11); lymphocytes 1 (1.23±0.16); monocytes 0.4 (0.41±0.19); epithelial cells 2 (2.17±0.41); goblet cells 0.2 (0.20±0.04) [5,7,8,29,30,56].

**Table 8:** Mean numbers of particular cell types in tears (per microscopic field at 250×magnification) during the positive SDYCR and PBS challenge (n=8).

Cells	After the challenge (h)													
	Before the challenge	1	4	8	12	24	26	28	30	32	34	36	48	56
**Eosinophils**														
-SICR	1	0.5	1	0	0	0	0	0	0	0	0	0	0	0
-PBS	0	0	0.3	0	0	0	0	0	0	0	0	0	0	0
**Neutrophils**														
-SICR	0	0	0	0	3*	4.5*	4.5*	3*	3*	2*	0.5	0	0	0
-PBS	0	0	0	0.2	0	0	0	0	0	0	0	0.2	0	0
**Basophils**														
-SICR	0	0.1	0	0	0	0	0	0	0	0	0	0	0	0
-PBS	0	0	0	0	0	0	0	0	0	0	0	0	0	0
**Mast cells**														
-SICR	0	0	0	0	0	0	0	0	0	0	0	0	0	0
-PBS	0	0	0	0	0	0	0	0	0	0	0	0	0	0
**Lymphocytes**														
-SICR	0	0	0.1	0	0	3*	5*	4*	4.5*	3.5*	2*	0.4	0	0
-PBS	0	0	0	0.2	0	0	0	0	0	0	0	0	0	0
**Monocytes**														
-SICR	0	0	0	0	1*	2.5*	2*	2*	1.5*	0.2	0	0	0	0
-PBS	0	0	0	0	0	0	0	0	0	0	0	0	0	0
**Goblet cells**														
-SICR	0	0	0	0	0	0	0	0	0	0.1	1*	1*	0	0
-PBS	0	0	0	0	0	0	0	0	0	0	0	0	0	0
**Epithelial cells**														
-SICR	0.5	0	0	0	0.4	0	3*	6*	4.5*	2.5*	2*	0	0	0
-PBS	0	0.2	0	0	0	0	0	0	0	0	0	0	0	0

SDYCR=secondary delayed conjunctival response; PBS=phosphate-buffered saline; Significance with respect to the baseline (before the challenge): ^+^=p<*±0.05;* *=p<*0.05*; The statistically significant magnitude of changes in the cells in tears (mean ± SD), were as follows: eosinophils 2 (2.10±0.14); neutrophils 2 (2.25±0.21); basophils 0.3 (0.28±0.04); mast cells 0.5 (0.35±0.11); lymphocytes 1 (1.23±0.16); monocytes 0.4 (0.41±0.19); epithelial cells 2 (2.17±0.41); goblet cells 0.2 (0.20±0.04) [5,7,8,29,30,56].

### Control patients

The ten control subjects demonstrated neither conjunctival signs nor significant concentration changes in the factors in tears during the ten repeated positive nasal responses (p>0.2).

## Discussion

The conjunctiva and the nose have a manifold anatomic and functional relationship [2,3,7-10]. The conjunctiva communicates with the nasal cavity through the nasolacrimal duct facilitating tear drainage into the nasal cavity, and through the blood vessel, lymphatic, and neurogenic networks.

Allergic reactions taking place primarily in the nasal mucosa due to intranasal exposure to an inhalant allergen may affect the conjunctiva and other ocular tissues, such as the cornea, in various ways and upon involvement of various mechanisms [7-10,20,31-34]. These mechanisms may include the following: (1) Various cell types participating in the nasal allergic reaction can migrate into the bloodstream and/or lymphatic system and sometimes into lacrimal ways, and thus attain the conjunctiva. (2) Various factors (classical mediators, eicosanoids, cytokines, chemokines, adhesion molecules, and other factors) released during the allergic reaction in the nasal mucosa can reach the conjunctiva either directly by retrograde penetration through the lacrimal ways or indirectly through the related blood or lymphatic vessel system. (3)The nasal allergic reaction can also activate the local neurogenic system (sensory nerves, sympathetic and parasympathetic fibers) releasing the neuropeptides that reach the conjunctiva through related nerves, such as the nervus trigemini, nervus nasociliaris, and ganglion pterygopalatinum. (4) The nasal allergic reaction can also stimulate the local nasal mucosal lymphatic system called the nose-associated lymphatic tissue (NALT), a part of the mucosa-associated lymphatic system (MALT). The MALT system facilitates multiple mutual communication among the particular lymphatic organ-related sub-systems, such as the NALT and the eye-associated lymphatic tissue (EALT), conjunctiva-associated lymphatic tissue (CALT), tear-associated lymphatic tissue (TALT), and lacrimal drainage-associated lymphatic tissue (LDALT). The manifold communication among the individual lymphatic system parts results in transmission of various cell-cell, cell-receptor, and receptor-receptor signals as well as reciprocal traffic of various circulating cell types, such as plasma cells/B lymphocytes producing immunoglobulins of individual classes and sub-classes, particular sub-sets of T-lymphocytes (Th1- and Th2-cells, cytotoxic, regulatory, and natural killer cells), antigen-presenting cells (APCs), and other cell types [8-10,20,29-32]. The cell traffic can be realized not only through various attraction mechanisms governed by chemotactic factors, cytokines, chemokines, and adhesion molecules but also through the homing mechanism of B- and T-lymphocytes, controlled by several homing factors [8-10,20,31-34]. The disturbed homing mechanism leads to migration of particular cell types (e.g., B- or T-lymphocytes) to locations different from the predetermined destinations. The particular lymphocyte sub-sets having been initially activated in a certain tissue, after migrating into the blood and/or lymphatic stream to finish their maturation process, do not return to this original tissue, but terminate their route in another tissue. This process is called wrong homing [8-10,20,31-34].

The occurrence and possible role of various mediators and cytokines in patients with various forms of allergic conjunctivitis has already been extensively investigated [11-21,23,33,35-54]. The frequently studied mediators in tears included histamine, tryptase, ECP, LTB_4_, LTC_4_, MPO, cytokines, such as IL-1β, IL-2, IL-4, IL-5, IL-6, IL-10, IL-12p70, IL-13, IFN-γ, tumor necrosis alpha (TNF-α), and chemokines, such as monokine-induced by gamma interferon (MIG),interferon gamma-induced protein ( IP-10), interferon inducible T-cell alpha chemoattractans ( I-TAC), regulated on activation normal T cell expressed and secreted (RANTES) and eotaxins. In most of these studies, a single determination of these factors was performed in the tears of patients with the primary forms of allergic conjunctivitis or keratoconjunctivitis. Studies following the concentration changes in mediators in tears during particular types of conjunctival response, such as immediate or late CR, due to the conjunctival provocation tests with allergens (CPTs), are not numerous [11,15,35,36,38-40,45,52,54,55]. Although the primary form of a delayed CR has not yet been reported, the author’s preliminary, not yet published, data suggest the existence of such a type.

The primary form of immediate CR has been reported to be accompanied by increased concentration of histamine, tryptase, ECP, LTB_4_, LTC_4_, MPO, PGD_2_, and TAME-esterase in tears [11,15,18,35,38-40,44,45,52,54,55]. The primary form of late CR has been reported to be associated with increased concentrations of histamine, ECP, LTB_4_, and LTC_4_ in tears [11,12,15,35,36,45,52]. Although the primary and the secondary induced forms CR result in similar clinical features, the pathways and underlying immunologic mechanisms are different [1-11,16,19,35,56,57]. The most marked differences between the CR response forms concern the appearance of individual cell types and particular mediators in tears accompanying the particular CR forms. The SICRs and SLCRs are associated with distinctly lower counts of all cell types and lower concentrations of mediators in tears than the corresponding primary CR types due to the CPT [7,10,11,15,17,18,23,44,56]. In contrast to the other investigators’ findings concerning the primary CR forms, no MPO and LTB_4_ were recorded in tears during SICRs, whereas MPO has been detected in tears during SLCRs [11,15,35,38,44]. These results suggest the involvement of neutrophils in the mechanism underlying the SLCR but not in that leading to the SICR.

Generally, there is a dearth of structural data demonstrating the course of mediator and cytokine profiles in tears during the particular types of the primary conjunctival response to the CPT. Data illustrating the appearance and concentration changes of mediators and cytokines in tears during the secondary forms of conjunctival response to NPTs are, unfortunately, not available. Moreover, few data are also available on the mediator and cytokine appearance and profiles in the tears of healthy subjects, which may serve as reference values [58,59].

Our results demonstrating increased concentrations of histamine, tryptase, ECP, LTC_4,_ IL-4, and IL-5 in tears during the SICR would suggest the involvement of mast cells, eosinophils, and probably of Th_2_-lymphocytes in the mechanism underlying this response type. The concentration changes in histamine, ECP, LTB_4_, LTC_4_, MPO, IL-4, and IL-5 in tears during the SLCR may be suggestive of the role of basophils, eosinophils, neutrophils, and Th_2_-lymphocytes in SLCR, whereas the concentration changes of LTB_4_, MPO, IFN-γ, and IL-2 in tears during the SDYCR may be indicative of the involvement of neutrophils, monocytes, and Th_1_-lymphocytes in this response type. However, this presumption has also some limitations since various mediators and cytokines can be produced by various cell types. Moreover, these mediators have most probably been released by cells participating in the primary allergic reactions in the nasal mucosa. This hypothesis is supported by our previous results, demonstrating only limited numbers of these cell types, in a non-activated condition, in tears during the secondary conjunctival response types [7-10,23,56].

Another important aspect concerns the participation of the cells in an immunologic process. The appearance of a certain cell type in a medium, in this case in the tears, does not necessarily signify its activation and active participation in that immunologic process but can also be a consequence of another, proceeding, mechanism or its part. Vice versa, the activation of a particular cell type and its active participation in an immunologic process may not necessarily result in the changes of its count (e.g., increase).

The simultaneous recording of the concentration changes of particular mediators and some cytokines in the tears and of the changes in the counts of individual cell types in the tears, performed in the current study, has revealed activation and active participation of the relevant cell types in the mechanism(s) underlying the particular SCR types. The active participation of these cells in the particular SCR can be summarized as follows: (a) SICR: mast cells (histamine, tryptase), eosinophils (ECP, LTC_4_), Th_2_-lymphocytes (IL-4, IL-5); (b) SLCR: basophils (histamine), eosinophils (ECP, LTC_4_), neutrophils (MPO, LTB_4_), Th_2_-lymphocytes (IL-4, IL-5); (c) SDYCR: neutrophils (MPO, LTB_4_), Th_1_-lymphocytes (IL-2, IFN-γ), and monocytes (LTB_4_) [60]. The active participation of the individual cell types in the mechanism(s) underlying the particular SCR types has led to the changes in their counts and to the concentration changes of their constituents (mediators and some cytokines). The results of the current study confirm the cytologic profiles recorded in tears during the particular SCR types in our previous studies [7,10,23,56,61].

However, the results of the current study are limited only to the tears and cannot serve as an indication of the cellular and mediator changes and processes in the conjunctival mucosal membrane. These changes can be demonstrated only by biopsies [11,14,16]. This fact may be regarded as a limitation of the current study. The lack of measurements of the particular mediators and other cytokines in the nasal secretions simultaneously to their recording in the tears, which had not been performed due to technical reasons, is also a limitation of this study. In addition, the variation of the tear sample volumes may be considered as a certain deficit of this study. The results of the current study may therefore be assessed more as a trend of the individual mediator profiles in tears during the particular types of the secondary conjunctival response. A simultaneous measurement of some tear components, such as total protein, lysozyme or lactoferrin, indicating the tear dilution degree, would improve the quantification of investigated factors in tears in further studies. Similarly to all studies in tears, the exact differentiation of the basal portion of tears resulting from the immunologic mechanism(s) from the tear portion caused by the reflex mechanism during collection of the material is a difficult if not impossible technical problem. Nevertheless, we believe that the tear samples collected by a careful and non-irritating technique used in the current study, have been produced by the genuine immunologic mechanism. Even if some negligible reflex mechanism, in despite of gentle tear collection, has induced a marginal amount of reflex tears, then it was of the same range throughout the whole study, and thus without further significant influence on the study results.

The results of this study additionally stress the importance of provocation tests with allergens. The CPTs, performed directly on the conjunctiva, confirm the role of allergic reaction taking place in the conjunctiva due to the direct exposure of conjunctival tissue to an inhalant allergen [11-15,17-19,22-27,33,36,38-40,44,45,52,54,55]. The CPTs result in the manifestation of various types of primary conjunctival response characterized by typical ocular signs and subjective symptoms. The CPTs are therefore suitable for demonstrating the primary types of CR [11-15,17-19,22-27,33,36,38-40,44,45,52,54,55]. Conversely, the secondarily induced CR types can only be demonstrated by means of nasal provocation tests with allergens (NPTs) combined with simultaneous registration of the ocular signs and subjective symptoms. An important requirement for the CPTs and NPTs is registration of the particular representative parameters before and repeatedly after the allergen challenge for a sufficiently long period of time, allowing them to follow the dynamic course of the particular response type.

Finally, the results of the current study may also have an important impact on the therapeutic management of these ocular disorders, regarding not only the choice of the most suitable drugs but also the administration route [5-7,10,23,61-63]. However, further investigations, such as biopsy combined with immunohistochemical methods and flow cytometry of the conjunctival and adjacent tissues, will be necessary to clarify fully the mechanism(s) underlying the primary as well as the secondary conjunctival response types.

## References

[1] Bielory L, Friedlaender MH (2008). Allergic conjunctivitis.. Immunol Allergy Clin North Am.

[2] Barney NP, Graziano FM, Cook EB, Stahl JL. Allergic and immunologic diseases of the eye. In: Adkinson NF, Bochner BS, JW, Busse WW, Holgate ST, Lemanske RF, Simons FE, eds. Middleton’s Allergy, principles & practice (7th Ed). Philadelphia: Mosby –Elsevier Inc 2009: 1117–1137.

[3] Gurbaxani A, Calder VL, Lightman S. Ocular Allergy. In: Kay AB, Kaplan AP, Bousquet J, Holt P (Eds). Allergy and Allergic diseases. 2^nd^ Ed. Oxford: Willey Blackwell Publ Ltd, 2008; 1482–1509.

[4] Bielory L (2000). Allergic and immunologic disorders of the eye; Part I: Immunology of the eye; Part II: Ocular allergy.. J Allergy Clin Immunol.

[5] Pelikan Z (2002). The causal role of the nasal allergy in some patients with allergic conjunctivitis.. Allergy.

[6] Pelikan Z. Late nasal response-its clinical characteristics, features, and possible mechanisms. In: Dorsch W (Ed). Late Phase Allergic Reactions. Boca Raton, Ann Arbor, Boston (USA): CRC Press 1990: 111–155.

[7] Pelikan Z. The late nasal response. Thesis. Amsterdam: The Free University of Amsterdam 1996.

[8] Pelikan Z (2009). Seasonal and perennial allergic conjunctivitis: the possible role of nasal allergy.. Clin Experiment Ophthalmol.

[9] Pelikan Z (2009). The possible involvement of nasal allergy in allergic keratoconjunctivitis.. Eye (Lond).

[10] Pelikan Z (2010). Allergic conjunctivitis and nasal allergy.. Curr Allergy Asthma Rep.

[11] Bacon AS, Ahluwalia P, Irani AM, Schwartz LB, Holgate ST, Church MK, McGill JI (2000). Tear and conjunctival changes during the allergen-induced early- and late-phase responses.. J Allergy Clin Immunol.

[12] Leonardi A, Fregona IA, Plebani M, Secchi AG, Calder VL (2006). Th1- and Th2-type cytokines in chronic ocular allergy.. Graefes Arch Clin Exp Ophthalmol.

[13] Leonardi A, Curnow SJ, Zhan H, Calder VL (2006). Multiple cytokines in human tear specimens in seasonal and chronic allergic eye disease and in conjunctival fibroblast cultures.. Clin Exp Allergy.

[14] Metz DP, Hingorani M, Calder VL, Buckley RJ, Lightman SL (1997). T-cell cytokines in chronic allergic eye disease.. J Allergy Clin Immunol.

[15] Bonini S, Bonini S, Berruto A, Tomassini M, Carlesimo S, Bucci MG, Balsano F (1989). Conjunctival provocation test as a model for the study of allergy and inflammation in humans.. Int Arch Allergy Appl Immunol.

[16] Leonardi A, De Dominics C, Motterle L (2007). Immunopathogenesis of ocular allergy: a schematic approach to different clinical entities.. Curr Opin Allergy Clin Immunol.

[17] Anderson DF (1996). The conjunctival late-phase reaction and allergen provocation in the eye.. Clin Exp Allergy.

[18] Leonardi A (2005). In-vivo diagnostic measurements of ocular inflammation.. Curr Opin Allergy Clin Immunol.

[19] Choi SH, Bielory L (2008). Late-phase reaction in ocular allergy.. Curr Opin Allergy Clin Immunol.

[20] Dua HS, Gomes JA, Donoso LA, Laibson PR (1995). The ocular surface as part of the mucosal immune system: conjunctival mucosa-specific lymphocytes in ocular surface pathology.. Eye (Lond).

[21] Cook EB (2004). Tear cytokines in acute and chronic ocular allergic inflammation.. Curr Opin Allergy Clin Immunol.

[22] Friedlaender MH (2003). Conjunctival provocation testing: Overview of recent clinical trials in ocular allergy.. Int Ophthalmol Clin.

[23] Pelikan Z. Cytologic changes in tears during the late type of secondary conjunctival response induced by nasal allergy. In: Pelikan Z (Ed). Conjunctivitis- A complex and multifaceted disorder. Rijeka (Croatia): InTech 2011; 75–92–15-

[24] Melillo G, Bonini S, Cocco G, Davies RJ, De Monchy JGR, Frølund L, Pelikan Z (1997). Provocation tests with allergens.. Allergy.

[25] Abelson MB, Chambers WA, Smith LM (1990). Conjunctival allergen challenge. A clinical approach to studying allergic conjunctivitis.. Arch Ophthalmol.

[26] Abelson M, Howes J, George M (1998). The conjunctival provocation test model of ocular allergy: Utility for assessment of an ocular corticosteroid, Loteprednol etabonate.. J Ocul Pharmacol Ther.

[27] Abelson MB, George MA, Schaefer K, Smith LM (1994). Evaluation of the new ophthalmic antihistamine, 0.05% levocabastine in the clinical allergen challenge model of allergic conjunctivitis.. J Allergy Clin Immunol.

[28] Siraganian RP. Histamine release and assay methods for the study of human allergy. In: Rose NR, Friedman H, Fahey JL, Eds. Manual of clinical laboratory immunology. 3^rd^ Ed. Washington (DC): American Society of Microbiology 1986; 675–684.

[29] Pelikan Z, Pelikan-Filipek M (1988). Cytologic changes in the nasal secretions during the immediate nasal response.. J Allergy Clin Immunol.

[30] Pelikan Z, Pelikan-Filipek M (1989). Cytologic changes in the nasal secretion during the late nasal response.. J Allergy Clin Immunol.

[31] O’Sullivan NL, Montgomery PC, Sullivan DA. Ocular mucosal immunity. In: Mestecky J, Binnenstock J, Lamm M, Strober W, McGhee J, Mayer L (eds). Mucosal immunology (3rd Ed). Burlington (MA,USA), San Diego (CA,USA), London: Elsevier- Academic Press 2005: 1477–1496.

[32] Youngman KR, Lazarus NH, Butcher EC. Lymphocyte homing: Chemokines and adhesion molecules in T cell and IgA plasma cell localization in the mucosal immune system. In: Mestecky J, Binnenstock J, Lamm M, Strober W, McGhee J, Mayer L (eds). Mucosal immunology (3rd). Burlington (MA,USA), San Diego (CA,USA), London:Elsevier-Academic Press 2005: 667–680.

[33] Sacchetti M, Micera A, Lambiase A, Speranza S, Mantelli F, Petrachi G, Bonini S, Bonini S (2011). Tear levels of neuropeptides increase after specific allergen challenge in allergic conjunctivitis.. Mol Vis.

[34] Zoukhri D (2006). Effect of inflammation on lacrimal gland function.. Exp Eye Res.

[35] Leonardi A, Borghesan F, Faggian D, DePaoli M, Secchi AG, Plebani M (2000). Tear and serum soluble leukocyte activation markers in conjunctival allergic diseases.. Am J Ophthalmol.

[36] Montan PG (1996). Hage-Hamsteren van M, Zetterström O. Sustained eosinophil cationic protein release into tears after a single high-dose conjunctival allergen challenge.. Clin Exp Allergy.

[37] Tabbara KF (2001). Tear tryptase in vernal keratoconjunctivitis.. Arch Ophthalmol.

[38] Leonardi A, Busato F, Fregona I, Plebani M, Secchi AG (2000). Anti-inflammatory and antiallergic effects of ketorolac trimethamine in the conjunctival provocation model.. Br J Ophthalmol.

[39] Mita H, Sakuma Y, Shida T, Akiyama K (1994). Release of chemical mediators in the conjunctival lavage fluids after eye provocation with allergen or compound 48/80.. Arerugi.

[40] Kari O, Salo OP, Halmepuro L, Suvilehto K (1985). Tear histamine during allergic conjunctivitis challenge.. Graefes Arch Clin Exp Ophthalmol.

[41] Helintö M, Renkonen R, Tervo T, Vesaluoma M (2004). Saaren-Seppälä, Haahtela T, Kirveskari J. Direct in vivo monitoring of acute allergic reactions in human conjunctiva.. J Immunol.

[42] Oh JW, Shin JC, Jang SJ, Lee HB (1999). Expression of ICAM-1 on conjunctival epithelium and ECP in tears and serum of children with allergic conjunctivitis.. Ann Allergy Asthma Immunol.

[43] Nathan H, Naveh N, Meyer E (1994). Levels of prostaglandin E2 and leukotriene B4 in tears of vernal conjunctivitis patients during a therapeutical trial with indomethacin.. Doc Ophthalmol.

[44] Bisgaard H, Ford-Hutchinson AW, Charleson S, Taudorf E (1985). Production of leukotrienes in human skin and conjunctival mucosa after specific allergen challenge.. Allergy.

[45] Leonardi AA, Smith LM, Fregona IA, Salmaso M, Secchi AG (1996). Tear histamine and histaminase during the early (EPR) and late-16- (LPR) phases of the allergic reaction and the effects of lodoxamide.. Eur J Ophthalmol.

[46] Leonardi A, Sathe S, Bartolotti M, Beaton A, Sack R (2009). Cytokines, matrix metalloproteases, angiogenic and growth factors in tears of normal subjects and vernal keratoconjunctivitis patients.. Allergy.

[47] Uchio E, Ono SY, Ikezawa Z, Ohno S (2000). Tear levels of interferon-gamma, interleukin (IL)-2, IL-4 and IL-5 in patients with vernal keratoconjunctivitis, atopic keratoconjunctivitis and allergic conjunctivitis.. Clin Exp Allergy.

[48] Sack RA, Conradi L, Krumholz D, Beaton A, Sathe S, Morris C (2005). Membrane array characterization of 80 chemokines, cytokines, and growth factors in open- and closed-eye tears: angiogenin and other defense system constituents.. Invest Ophthalmol Vis Sci.

[49] Leonardi A, Borghesan F, Faggian D, Secchi A, Plebani M (1995). Eosinophil cationic protein in tears of normal subjects and patients affected by vernal keratoconjunctivitis.. Allergy.

[50] Bonini S, Lambiase A, Sacchetti M, Bonini S (2003). Cytokines in ocular allergy.. Int Ophthalmol Clin.

[51] Calder VL, Jolly G, Hingorani M, Adamson P, Leonardi A, Secchi AG, Buckley RJ, Lighman S (1999). Cytokine production and mRNA expression by conjuctival T-cell lines in chronic allergic eye disease.. Clin Exp Allergy.

[52] Ahluwalia P, Anderson DF, Wilson SJ, McGill JI, Church MK (2001). Nedocromil sodium and levocabastine reduce the symptoms of conjunctival allergen challenge by different mechanisms.. J Allergy Clin Immunol.

[53] Magrini L, Bonini S, Centofanti M, Schiavone M, Bonini S (1996). Tear tryptase levels and allergic conjunctivitis.. Allergy.

[54] Proud D, Sweet J, Stein P, Settipane RA, Kagey-Sobotka A, Friedlaender MH, Lichtenstein LM (1990). Inflammatory mediator release on conjunctival provocation of allergic subjects with allergen.. J Allergy Clin Immunol.

[55] Friedlaender MH (1989). Conjunctival provocative tests: A model of human ocular allergy.. Trans Am Ophthalmol Soc.

[56] Pelikan Z (2012). Cytologic changes in tears during the secondary conjunctival response induced by nasal allergy.. Br J Ophthalmol.

[57] Ono SJ, Abelson MB (2005). Allergic conjunctivitis: Update on pathophysiology and prospects for future treatment.. J Allergy Clin Immunol.

[58] Nakamura Y, Sotozono C, Kinoshita S (1998). Inflammatory cytokines in normal human tears.. Curr Eye Res.

[59] Carreño E, Enriquez-de Salamanca A, Teson M, Garcia-Vazquez C, Stern ME, Whitcup SM, Calonge M (2010). Cytokine and chemokine levels in tears from healthy subjects.. Acta Ophthalmol (Copenh).

[60] Rankin JA, Lee TH. Monocytes and macrophages. In: Middleton E, Reed ChE, Ellis EF, Adkinson NF, Yunginger JW, Busse WW (Eds). Allergy, principles and practice. 4^th^ Ed. St.Louis, Baltimore, Boston, Chicago, London, Philadelphia, Sydney, Toronto: Mosby-Year Book Inc 1993; 226–242.

[61] Pelikan Z, Pelikan M (1985). The role of the nasal mucosa in some cases of allergic conjunctivitis and the effects of disodium cromoglycate (DSCG).. J Allergy Clin Immunol.

[62] Pelikan Z (1988). Allergic conjunctivitis: primary and secondary role of the allergy reaction in the nose.. Dutch Journal of Medicine.

[63] Pelikan Z. Anti-allergic drugs and immunotherapy. In: Principles of Immunopharmacology. Nijkamp FP, Parnham MJ (Eds) Basel, Boston, Berlin: Birkhäuser Verlag, 1999; 243–268.

